# Supplementary material for: The association between depression and later educational attainment in children and adolescents: a systematic review protocol
Source: BMJ Open. 2019 Nov 14;9(11):e031595. doi: 10.1136/bmjopen-2019-031595 (PMC6886932; doi:10.1136/bmjopen-2019-031595)
Supplement: Supplementary data [file bmjopen-2019-031595supp002.pdf]

Supplementary file 2 – Data extraction form

- Lead author
- Year
- Country
- Participant inclusion/exclusion criteria
- Sample size for reported analysis
- Sample type (clinical/community/school)
- Age at exposure (mean, median and/or range)
- Follow-up period
- Gender balance
- Exposure type (binary/continuous/categorical)
- Exposure method of ascertainment
- Outcome type (binary/continuous/categorical)
- Outcome method of ascertainment
- Multivariable or bivariate effect estimate
- Confidence intervals or standard error
- p value
- Covariates and confounders adjusted for
- Moderators reported on
- Moderator findings
- Mediators reported on
- Mediator findings

Wickersham A, *et al. BMJ Open* 2019; 9:e031595. doi: 10.1136/bmjopen-2019-031595
