# Supplementary material for: The association between depression and later educational attainment in children and adolescents: a systematic review protocol
Source: BMJ Open. 2019 Nov 14;9(11):e031595. doi: 10.1136/bmjopen-2019-031595 (PMC6886932; doi:10.1136/bmjopen-2019-031595)
Supplement: Author's manuscript [file bmjopen-2019-031595.draft_revisions.pdf]

BMJ Open is committed to open peer review. As part of this commitment we make the peer review history of every article we publish publicly available.

When an article is published we post the peer reviewers' comments and the authors' responses online. We also post the versions of the paper that were used during peer review. These are the versions that the peer review comments apply to.

The versions of the paper that follow are the versions that were submitted during the peer review process. They are not the versions of record or the final published versions. They should not be cited or distributed as the published version of this manuscript.

BMJ Open is an open access journal and the full, final, typeset and author-corrected version of record of the manuscript is available on our site with no access controls, subscription charges or pay-per-view fees (http://bmjopen.bmj.com).

If you have any questions on BMJ Open's open peer review process please email info.bmjopen@bmj.com

BMJ Open

The association between depression and later educational attainment in children and adolescents: A systematic review protocol

Journal:	*BMJ Open*
Manuscript ID	bmjopen-2019-031595
Article Type:	Protocol
Date Submitted by the Author:	10-May-2019
Complete List of Authors:	Wickersham, Alice; King's College London, Institute of Psychiatry, Psychology and Neuroscience;<br>Epstein, Sophie; South London and Maudsley NHS Foundation Trust, NIHR Maudsley Biomedical Research Centre<br>Sugg, Holly; University of Exeter, Medical School<br>Stewart, Robert; King's College London, Institute of Psychiatry<br>Ford, Tamsin; University of Exeter<br>Downs, Johnny; Kings College London, Institute of Psychiatry, Psychology and Neuroscience
Keywords:	Child & adolescent psychiatry < PSYCHIATRY, Depression & mood disorders < PSYCHIATRY, EPIDEMIOLOGY, EDUCATION & TRAINING (see Medical Education & Training)

SCHOLARONE™
Manuscripts

The association between depression and later educational attainment in children and adolescents: A systematic review protocol

Alice Wickersham[1,2], Sophie Epstein[1,2], Holly VR Sugg[3], Robert Stewart[1,2], Tamsin Ford[3] & Johnny Downs[1,2]

[1] Institute of Psychiatry, Psychology & Neuroscience, King's College London, London, UK

[2] NIHR Maudsley Biomedical Research Centre, South London and Maudsley NHS Foundation Trust, London, UK

[3] University of Exeter Medical School, Exeter, UK

Correspondence to:

Alice Wickersham

ORCID ID: 0000-0002-7402-7690

Institute of Psychiatry, Psychology and Neuroscience, King's College London, 16 De Crespigny Park, London, SE5 8AF

alice.wickersham@kcl.ac.uk

020 7848 0002

Word count: 2437 (excluding title page, abstract, references, figures, tables and acknowledgements)

For peer review only

Abstract

Introduction: Depression represents a major public health concern for children and adolescents, and is thought to negatively impact subsequent educational attainment. However, the extent to which depression and educational attainment are directly associated, and whether other factors play a role, is uncertain. Therefore we aim to systematically review the literature to provide an up-to-date estimate on the strength of this association, and to summarise potential mediators and moderators on the pathway between the two.

Methods and analysis: To identify relevant studies we will systematically search Embase, PsycINFO, PubMed, Education Resources Information Centre and British Education Index, manually search reference lists and contact experts in the field. Studies will be included if they investigate and report on the association between major depression diagnosis or depressive symptoms in children and adolescents aged 4 to 18 years (exposure) and later educational attainment (outcome). Two independent reviewers will screen titles, abstracts and full texts according to eligibility criteria, perform data extraction, and assess study quality according to a modified version of the Newcastle-Ottawa Scale. If sufficiently homogenous studies are identified, summary effect estimates will be pooled in meta-analysis, with further tests for study heterogeneity, publication bias, and the effects of moderators using meta-regression.

Ethics and dissemination: Because this review will make use of already published data, ethical approval will not be sought. The review will be submitted for publication in a peer-reviewed journal, presented at practitioner-facing conferences, and a lay summary will be written for non-scientific audiences such as parents, young people and teachers. The work will inform upcoming investigations on the association between child and adolescent mental health and educational attainment.

PROSPERO registration number: CRD42019123068

Key words: Child & adolescent psychiatry; Depression & mood disorders; Epidemiology; Education & training

ARTICLE SUMMARY

Strengths and limitations of this study

- This review will provide a timely update on the association between depression and later educational attainment, and will be the first to summarise evidence on both moderators and mediators of the association.
- A comprehensive search strategy is planned, including searches of both health and education electronic databases, forward and backward citation searching, and contacting authors and experts in the field.
- The exclusion of grey literature and studies not published in the English language may cause some relevant studies to be missed.
- However, limiting the review to peer-reviewed studies which make use of standardised depression measures and academic records will ensure that included studies are of a reasonable quality, and reduce heterogeneity for meta-analysis.
- Including prospective longitudinal studies will also aid inference of a causal direction between depression and later educational attainment.

For peer review only - http://bmjopen.bmj.com/site/about/guidelines.xhtml

INTRODUCTION

Depression is a major public health concern for young people, and appears to be on the rise, with a recent study estimating that 2.1% of children and adolescents in England meet criteria for a depressive disorder. [1-3] Depression can impact educational performance; a meta-analysis of longitudinal studies by Riglin et al. found that depression was associated with the failure to complete compulsory education and low school grades. [4] Poor school performance can in turn have far-reaching negative consequences during adulthood, predicting unemployment, homelessness, poor health, and suicide attempt. [5-8]

At the time of Riglin et al.'s review, [4] comparatively few studies had been carried out outside of North America. Additionally, while the presence of depressive symptoms remains an exposure of interest, Riglin et al. were unable to draw comparisons of studies to examine the effect of clinical depression on grades, due to an insufficient number of such studies being available. These gaps in the literature may have since been addressed. Therefore, the first aim of our systematic review is to revisit this association and provide an up-to-date estimate on the strength of the relationship between child and adolescent depression and later educational attainment.

Beyond an examination of the moderating effects of age, gender, and length of study follow-up period, Riglin et al. considered evidence on the pathways between depression and educational attainment to be beyond the scope of their systematic review. They suggested that mechanisms at the participant- and school-level should be further investigated. Indeed, a range of candidate factors are thought to affect school performance and may conceivably mediate or moderate the impact of depression, such as executive function, sleep, classroom environment and engagement, family environment, and parent involvement. [9-13] Therefore, the second aim of our systematic review is to investigate mediators and moderators in the relationship between child and adolescent depression and later educational attainment, with the intention of proposing a pathway model between the two.

METHODS AND ANALYSIS

This protocol follows the PRISMA reporting guidelines for systematic review and meta-analysis protocols (supplementary file 1). [14] The final review will also follow PRISMA reporting guidelines, and will include a PRISMA checklist and flow diagram. [15] Details of the protocol for this systematic review were registered on PROSPERO and can be accessed at www.crd.york.ac.uk/PROSPERO/display_record.asp?ID=CRD42019123068. Any amendments to the protocol will be registered on PROSPERO.

Eligibility criteria

Table 1: Summary of inclusion and exclusion criteria

Inclusion criteria
• Studies which investigate and report results on the relationship between depression diagnosis or depressive symptoms (exposure) and later educational attainment (outcome)<br>• Studies where participants are aged 4-18 years (inclusive) at the time of exposure measurement<br>• Studies which make use of data from countries with compulsory education policies<br>• Quantitative longitudinal studies with prospective data collection<br>• Original research published in a peer-reviewed journal

For peer review only - http://bmjopen.bmj.com/site/about/guidelines.xhtml

• Published in English<br>• Full-text available including data on the association between depression and educational attainment
**Exclusion criteria**
• Studies which only investigate internalising symptoms or other affective disorders such as bipolar disorder<br>• Studies which recruit participants from post-secondary education settings<br>• Studies which do not use a standardised diagnostic measure or a named measurement instrument to measure depression or depressive symptoms<br>• Studies which do not measure educational attainment using academic or administrative records, or where the source is unclear<br>• Studies that only investigate school dropout, general intelligence, aptitude, or ability as the outcome of interest<br>• Studies aiming to conduct or evaluate an intervention during the observed study period

Population

Inclusion and exclusion criteria are summarised in Table 1. Participants in included studies will be children and adolescents, all within the 4 to 18 year age range at the time of exposure measurement, encompassing the compulsory school age range in most countries. Studies will make use of data from countries with compulsory educational policies – in countries without such policies, school attendance is likely to be poorer for children with mental health difficulties such as depression, and therefore attainment data is less likely to be adequately available for this group. [16] Studies will be excluded if they recruit participants from post-secondary education settings such as universities at baseline, to ensure that results pertain to depression in childhood and adolescence during school. Age at the time of outcome measurement will not be restricted, such that attainment at higher education will be included.

Exposure and outcome variables

Included studies will investigate and report results on the relationship between child or adolescent depression (exposure) and later educational attainment (outcome). Child or adolescent depression will be operationalised as depressive symptoms or depression diagnosis as identified using a standardised diagnostic measure or a named measurement instrument. Studies of internalising symptoms or other depressive disorders, such as bipolar disorder, will not be included. We anticipate that studies will use various measures of educational attainment, including but not limited to highest level of education completed, and standardised academic assessment scores. Studies will not be included if they do not measure educational attainment using academic or administrative records, instead relying on an unclear source or on self-, parent-, or teacher only-reported outcomes. The accuracy of informant-rated academic performance can be mixed compared to results from externally validated assessments. [17] School dropout will not be considered in this review as it represents a distinct construct which may have different mechanisms associated with it; indeed poor educational attainment is thought to be one of the predictors of school dropout. [18] Measures of general intelligence, aptitude, or ability will also not be included.

Types of studies

To aid the inference of a causal direction, only quantitative, prospective longitudinal studies will be included. Reviews, meta-analyses, cross-sectional studies, case reports, clinical vignettes, Randomised Controlled Trials, and exclusively qualitative studies will therefore be excluded. Studies

For peer review only

which aim to conduct or evaluate an intervention during the observed study period will also be excluded to ensure that any observed association was not influenced by the intervention. Studies will be included if they are original research in English, published in a peer-reviewed journal (therefore excluding grey literature, books, chapters, theses, dissertations, conference proceedings, editorials, opinion pieces, letters to the editor, and commentaries), and if the full-text is available (including data on the association between depression and educational attainment). Corresponding authors will be emailed for full-texts that cannot be obtained via institutional access. To maximise the number of relevant studies captured, no date range will be imposed on the search.

Information sources

Studies will be identified by searching the following electronic databases:

- Embase (via Ovid)
- PsycINFO (via Ovid)
- PubMed (via NCBI)
- Education Resources Information Centre (ERIC) (via EBESCO)
- British Education Index (via EBESCO)

We will also search reference lists of included studies and relevant existing reviews (backward citation searching), and papers which have referenced them (forward citation searching). Backward and forward citation searching will be carried out in Web of Science Core Collection. If a citation cannot be identified in Web of Science, Google Scholar will be searched. Finally, experts in the field and corresponding authors of included studies will be contacted to identify any additional papers.

Search strategy for electronic databases

Electronic database searching will be conducted using a combination of key words (using truncation as needed) and subject headings (exploded to include narrower terms). The exact search terms used will therefore be adapted according to database thesauruses, but broadly will be grouped according to three concepts:

1. Age (e.g. child, adolescent, youth)
2. Educational attainment (e.g. academic performance, educational attainment, school failure)
3. Depression (e.g. depression, depressive disorders)

English language limits will be applied. The full search strategy for Embase can be found in Table 2.

Table 2: Full search strategy for Embase

#	Search terms
1	exp adolescent/ OR exp adolescence/ OR exp child/ OR exp childhood/ OR child*.tw OR adolescen*.tw OR teenag*.tw OR youth*.tw OR (young adj (people or person)).tw
2	Limit 1 to english language
3	exp academic achievement/ OR exp outcome of education/ OR ((academic or educational or school or classroom) adj (achievement or performance or attainment or success or failure)).tw
4	Limit 3 to english language
5	exp depression/ OR depressi*.tw
6	Limit 5 to english language
7	#2 AND #4 AND #6

Note: exp [search term]/ denotes exploding a subject heading; .tw denotes searching for a key word in the title, abstract and drug trade name; * denotes truncation

For peer review only - http://bmjopen.bmj.com/site/about/guidelines.xhtml

Data management

All identified citations will be downloaded and managed in EndNote, and duplicates will be removed. Article screening and data extraction will be tracked using Microsoft Excel.

Selection process

Following electronic database searching, initial title and abstract screening will be carried out by two independent reviewers. The reviewers will initially screen 10% of the titles and abstracts and agreement will be checked, before proceeding to screen all the remaining titles and abstracts according to the pre-specified eligibility criteria (Table 1). All references will be screened by both reviewers to ascertain the level of agreement. Articles which appear eligible from the abstract, or are of unclear eligibility, will pass to full-text screening. This will also be carried out by two independent reviewers, again following an initial 10% screen to check agreement. The process of independent abstract and full-text screening will be repeated for references identified during backward and forward citation searching following an initial screen carried out by the lead researcher. Any disagreements over article eligibility will be discussed, and a third reviewer will be consulted if a consensus cannot be reached.

Data extraction

Data will be extracted using a data extraction form which will be informed by the full-text screening and will be piloted on the included studies before being finalised. The anticipated data extraction form is in supplementary file 2. Data extraction will be carried out by two reviewers. Any disagreements over data extraction will be discussed, and a third reviewer will be consulted if a consensus cannot be reached. If multiple studies use the same data sources, they will still be recorded separately in data extraction as they may offer insights to different covariates, mediators and moderators.

Quality assessment

The included studies will be assessed for risk of bias at the study-level using a modified version of the Newcastle-Ottawa Scale (NOS) for cohort studies (supplementary file 3). [19] The NOS is recommended by the Cochrane Handbook (section 13.5.2.3). [20] Some items have been adapted for relevance to this review (for instance making reference to depression, educational attainment, and schools), and two items on sample size and statistical tests have been added in accordance with a previous systematic review on education and mental health. [21] Risk of bias will be assessed by two independent reviewers. Disagreements will be discussed, and a third reviewer will be consulted if a consensus cannot be reached. Results from the risk of bias assessment will be taken into consideration when interpreting the strength of evidence for the reported associations, and will be considered in meta-analysis (see 'Data Synthesis').

Data synthesis

The characteristics and findings of included studies will be presented in a data extraction table and will be discussed in a narrative synthesis. If multiple studies are identified which investigate similar exposure and outcome variables, a random-effects meta-analysis will be conducted. If multiple articles measure the same association in an identical cohort, and if multiple relevant associations are reported within one article, the mean of these associations will be taken and used in meta-analysis.

Summary estimates for the effect of depression on later educational attainment will be pooled. Estimates from multivariable analyses which control for at least one covariate will be used, unless

For peer review only - http://bmjopen.bmj.com/site/about/guidelines.xhtml

insufficient studies conduct comparable multivariable analyses, in which case estimates from bivariate analyses will be used instead. A confidence interval and *p* value will be calculated for the pooled effect estimate. Heterogeneity of effect estimates will be investigated using the I^2 statistic. Publication bias will be assessed using funnel plots and Egger's test for publication bias if at least ten studies are included in the meta-analysis (Cochrane Handbook section 10.4.3.1). [20]

To investigate moderators in the association between depression and educational attainment, subgroup analyses will be carried out using meta-regression. As with Riglin et al., [4] we will examine the moderating effects of age, gender, and follow-up period. In addition, the impact of risk of bias will be investigated. Other investigations of potentially important moderators may be informed *post hoc* by the included studies, but will be identified as such in the final report as recommended in the Cochrane Handbook (section 9.6.5.2). [20] Candidate moderators include ethnicity, socio-economic status, co-morbidities, country, the measurement of clinical diagnosis versus symptomatology at exposure, and whether studies are conducted in clinical versus community settings. Finally, if multiple studies investigate similar mediator variables, meta-analytic structural equation modelling will be employed to synthesise their effects.

Study status

Electronic database searching was conducted in November 2018. The review is expected to be completed in July 2019.

ETHICS AND DISSEMINATION

This review will make use of already published data, therefore ethical approval will not be sought. On completion, the review will be submitted to a peer-reviewed journal in the field of mental health or educational research for publication. Findings will also be presented at practitioner-facing conferences, and a lay summary will be written for non-scientific audiences such as parents, young people and teachers. The findings will inform upcoming work on the association between child and adolescent mental health and educational attainment.

Patient and public involvement

This review was planned to support ongoing health and education data linkage work which has been carried out in consultation with several patient and caregiver groups at the National Institute for Health Research Biomedical Research Centre, South London and Maudsley NHS Foundation Trust and King's College London. [22 23] The results of the review and other research will be discussed with a young person's mental health advisory group, parents and teachers to guide upcoming work with linked health and education data.

DISCUSSION

While our methodology was informed by Riglin et al., [4] we have made several modifications to this design which we believe will strengthen the review. First, we will broaden our information sources to include Embase and British Education Index. Second, we will examine the 4 to 18 year age range rather than the 8 to 18 year age range, as this captures the compulsory school years in most countries. Third, we will not limit our review to community-based samples. While findings from clinical samples are of limited generalisability, this will maximise the amount of available evidence that is captured on the association, particularly with regard to diagnosed depressive disorders.

For peer review only - http://bmjopen.bmj.com/site/about/guidelines.xhtml

For peer review only

In our review we will focus on depression and will not report data on anxiety or other internalising disorders. Riglin et al. noted that the association between anxiety and educational attainment is much less clear, with the possibility that anxiety may sometimes have a positive role. Therefore, the pathways leading to educational attainment outcomes are likely to be different for anxiety and depression, and should be considered separately. Finally, to ensure the objectivity of our outcome measure, we will exclude studies which do not obtain educational attainment from academic or administrative records.

This review inevitably will have some limitations. The exclusion of studies not published in the English language may omit some studies of this association, however we lack resources for translation. Additionally, the exclusion of grey literature may also cause relevant findings to be missed, however peer-review was considered an important eligibility criterion to ensure a certain level of quality control in included studies.

This systematic review will provide a synthesis of the available evidence on the association between child and adolescent depression and later educational attainment. This work is timely and of great public interest. Recent studies demonstrate the high prevalence of mental health problems in the child and adolescent age group, [1-3] and ongoing plans to improve child mental health provision in schools demonstrate the increasing recognition that education and wellbeing are closely linked. [24] Understanding whether and how depression influences later educational attainment is critical to developing effective interventions for affected groups. To our knowledge this is the first systematic review to provide a focused synthesis on child and adolescent depression and academic records of educational attainment, with the additional aim of investigating mediators and moderators in order to propose a comprehensive pathway model on the association between the two.

Author contributions: AW (guarantor) co-conceived and designed the review and wrote the initial manuscript. SE advised on study design and provided revisions on the manuscript. HVRS provided revisions on the manuscript. RS, TF and JD co-conceived the review, advised on study design, and provided revisions on the manuscript.

Acknowledgements: With thanks to Simon Benham-Clarke and Darren Moore for providing comments on the search strategy.

Competing Interests: None declared

Funding statement: This paper represents independent research funded by the National Institute for Health Research (NIHR) Biomedical Research Centre at South London and Maudsley NHS Foundation Trust and King's College London. The views expressed are those of the authors and not necessarily those of the NHS, the NIHR or the Department of Health and Social Care. SE has been employed as an NIHR Academic Clinical Fellow and has received salary support from an MQ Data Science Award and from the Psychiatry Research Trust. HVRS is funded by the University of Exeter Medical School. JD is funded by an NIHR Clinician Scientist Award, and received support from a Medical Research Council (MRC) Clinical Research Training Fellowship (MR/L017105/1) and Psychiatry Research Trust Peggy Pollak Research Fellowship in Developmental Psychiatry.

Data Availability Statement: Not applicable

For peer review only - http://bmjopen.bmj.com/site/about/guidelines.xhtml

References

1. Lewis SJ, Arseneault L, Caspi A, et al. The epidemiology of trauma and post-traumatic stress disorder in a representative cohort of young people in England and Wales. *The Lancet Psychiatry* 2019;6(3):247-56. doi: 10.1016/S2215-0366(19)30031-8
2. Pitchforth J, Fahy K, Ford T, et al. Mental health and well-being trends among children and young people in the UK, 1995–2014: analysis of repeated cross-sectional national health surveys. *Psychological medicine* 2018:1-11.
3. Sadler K, Vizard T, Ford T, et al. Mental Health of Children and Young People in England, 2017. 2018
4. Riglin L, Petrides K, Frederickson N, et al. The relationship between emotional problems and subsequent school attainment: A meta-analysis. *Journal of adolescence* 2014;37(4):335-46.
5. Almquist YB. School performance as a precursor of adult health: exploring associations to disease-specific hospital care and their possible explanations. *Scandinavian journal of public health* 2013;41(1):81-91.
6. Brakenhoff B, Jang B, Slesnick N, et al. Longitudinal predictors of homelessness: Findings from the National Longitudinal Survey of Youth-97. *Journal of youth studies* 2015;18(8):1015-34.
7. Caspi A, Wright BRE, Moffitt TE, et al. Early failure in the labor market: Childhood and adolescent predictors of unemployment in the transition to adulthood. *American sociological review* 1998:424-51.
8. Sörberg Wallin A, Zeebari Z, Lager A, et al. Suicide attempt predicted by academic performance and childhood IQ: a cohort study of 26 000 children. *Acta psychiatrica scandinavica* 2018;137(4):277-86.
9. Dewald JF, Meijer AM, Oort FJ, et al. The influence of sleep quality, sleep duration and sleepiness on school performance in children and adolescents: a meta-analytic review. *Sleep medicine reviews* 2010;14(3):179-89.
10. Diamantopoulou S, Rydell A-M, Thorell LB, et al. Impact of executive functioning and symptoms of attention deficit hyperactivity disorder on children's peer relations and school performance. *Developmental neuropsychology* 2007;32(1):521-42.
11. Dotterer AM, Lowe K. Classroom context, school engagement, and academic achievement in early adolescence. *Journal of youth and adolescence* 2011;40(12):1649-60.
12. Marjoribanks K. Family environments and children's outcomes. *Educational Psychology* 2005;25(6):647-57.
13. Wilder S. Effects of parental involvement on academic achievement: a meta-synthesis. *Educational Review* 2014;66(3):377-97.
14. Shamseer L, Moher D, Clarke M, et al. Preferred reporting items for systematic review and meta-analysis protocols (PRISMA-P) 2015: elaboration and explanation. *Bmj* 2015;349:g7647.
15. Moher D, Liberati A, Tetzlaff J, et al. Preferred reporting items for systematic reviews and meta-analyses: the PRISMA statement. *Annals of internal medicine* 2009;151(4):264-69.
16. Quan A. Education for people and planet: Creating sustainable futures for all: Unesco 2016.
17. Kuncel NR, Credé M, Thomas LL. The validity of self-reported grade point averages, class ranks, and test scores: A meta-analysis and review of the literature. *Review of educational research* 2005;75(1):63-82.
18. De Witte K, Cabus S, Thyssen G, et al. A critical review of the literature on school dropout. *Educational Research Review* 2013;10:13-28.
19. Wells GA, Shea, B., O'Connell, D., Peterson, J., Welch, V., Losos, M., Tugwell, P. The Newcastle-Ottawa Scale (NOS) for assessing the quality of nonrandomised studies in meta-analyses 2008 [Available from: http://www.ohri.ca/programs/clinical_epidemiology/oxford.asp.
20. Higgins J, Green S. Cochrane Handbook for Systematic Reviews of Interventions Version 5.1.0 [updated March 2011]: The Cochrane Collaboration 2011.

For peer review only - http://bmjopen.bmj.com/site/about/guidelines.xhtml

21. Epstein S, Roberts E, Sedgwick R, et al. Poor school attendance and exclusion: a systematic review protocol on educational risk factors for self-harm and suicidal behaviours. *BMJ Open* 2018;8(12):e023953. doi: 10.1136/bmjopen-2018-023953
22. Downs J, Gilbert R, Hayes RD, et al. Linking health and education data to plan and evaluate services for children. *Archives of disease in childhood* 2017;102(7):599-602.
23. Downs JM, Ford T, Stewart R, et al. An approach to linking education, social care and electronic health records for children and young people in South London: a linkage study of child and adolescent mental health service data. *BMJ Open* 2019;9(1):e024355. doi: 10.1136/bmjopen-2018-024355
24. Department of Health and Social Care and Department for Education. Transforming children and young people's mental health provision: A green paper: DoH/DfE London, 2017.

For peer review only

For peer review only - http://bmjopen.bmj.com/site/about/guidelines.xhtml

Supplementary file 1 – PRISMA-P checklist

PRISMA-P (Preferred Reporting Items for Systematic review and Meta-Analysis Protocols) 2015 checklist: recommended items to address in a systematic review protocol*

Section and topic	Item No	Checklist item	Page number
**ADMINISTRATIVE INFORMATION**			
Title:			
Identification	1a	Identify the report as a protocol of a systematic review	1
Update	1b	If the protocol is for an update of a previous systematic review, identify as such	N/A
Registration	2	If registered, provide the name of the registry (such as PROSPERO) and registration number	1, 3
Authors:			
Contact	3a	Provide name, institutional affiliation, e-mail address of all protocol authors; provide physical mailing address of corresponding author	1
Contributions	3b	Describe contributions of protocol authors and identify the guarantor of the review	8
Amendments	4	If the protocol represents an amendment of a previously completed or published protocol, identify as such and list changes; otherwise, state plan for documenting important protocol amendments	N/A
Support:			
Sources	5a	Indicate sources of financial or other support for the review	8
Sponsor	5b	Provide name for the review funder and/or sponsor	8
Role of sponsor or funder	5c	Describe roles of funder(s), sponsor(s), and/or institution(s), if any, in developing the protocol	N/A
**INTRODUCTION**			
Rationale	6	Describe the rationale for the review in the context of what is already known	3
Objectives	7	Provide an explicit statement of the question(s) the review will address with reference to participants, interventions, comparators, and outcomes (PICO)	3
**METHODS**			
Eligibility criteria	8	Specify the study characteristics (such as PICO, study design, setting, time frame) and report characteristics (such as years considered, language, publication status) to be used as criteria for eligibility for the review	3, 4, 5

For peer review only

Information sources	9	Describe all intended information sources (such as electronic databases, contact with study authors, trial registers or other grey literature sources) with planned dates of coverage	5
Search strategy	10	Present draft of search strategy to be used for at least one electronic database, including planned limits, such that it could be repeated	5
Study records:			
Data management	11a	Describe the mechanism(s) that will be used to manage records and data throughout the review	6
Selection process	11b	State the process that will be used for selecting studies (such as two independent reviewers) through each phase of the review (that is, screening, eligibility and inclusion in meta-analysis)	6
Data collection process	11c	Describe planned method of extracting data from reports (such as piloting forms, done independently, in duplicate), any processes for obtaining and confirming data from investigators	6
Data items	12	List and define all variables for which data will be sought (such as PICO items, funding sources), any pre-planned data assumptions and simplifications	6, supplementary file 2
Outcomes and prioritization	13	List and define all outcomes for which data will be sought, including prioritization of main and additional outcomes, with rationale	4
Risk of bias in individual studies	14	Describe anticipated methods for assessing risk of bias of individual studies, including whether this will be done at the outcome or study level, or both; state how this information will be used in data synthesis	6
Data synthesis	15a	Describe criteria under which study data will be quantitatively synthesised	6, 7
	15b	If data are appropriate for quantitative synthesis, describe planned summary measures, methods of handling data and methods of combining data from studies, including any planned exploration of consistency (such as I^2, Kendall's τ)	6, 7
	15c	Describe any proposed additional analyses (such as sensitivity or subgroup analyses, meta-regression)	7
	15d	If quantitative synthesis is not appropriate, describe the type of summary planned	6
Meta-bias(es)	16	Specify any planned assessment of meta-bias(es) (such as publication bias across studies, selective reporting within studies)	7
Confidence in cumulative evidence	17	Describe how the strength of the body of evidence will be assessed (such as GRADE)	6, 7

*** It is strongly recommended that this checklist be read in conjunction with the PRISMA-P Explanation and Elaboration (cite when available) for important clarification on the items. Amendments to a review protocol should be tracked and dated. The copyright for PRISMA-P (including checklist) is held by the PRISMA-P Group and is distributed under a Creative Commons Attribution Licence 4.0.**

From: Shamseer L, Moher D, Clarke M, Ghersi D, Liberati A, Petticrew M, Shekelle P, Stewart L, PRISMA-P Group. Preferred reporting items for systematic review and meta-analysis protocols (PRISMA-P) 2015: elaboration and explanation. BMJ. 2015 Jan 2;349(jan02 1):g7647.

For peer review only

Supplementary file 2 – Data extraction form

- Lead author
- Year
- Country
- Participant inclusion/exclusion criteria
- Sample size for reported analysis
- Sample type (clinical/community/school)
- Age at exposure (mean, median and/or range)
- Follow-up period
- Gender balance
- Exposure type (binary/continuous/categorical)
- Exposure method of ascertainment
- Outcome type (binary/continuous/categorical)
- Outcome method of ascertainment
- Multivariable or bivariate effect estimate
- Confidence intervals or standard error
- p value
- Moderators reported on
- Moderator findings
- Mediators reported on
- Mediator findings

For peer review only

Supplementary file 3 – Modified Newcastle-Ottawa Quality Assessment Scale for cohort studies

Note: A study can be awarded a maximum of one star for each numbered item within the Selection and Outcome categories. A maximum of two stars can be given for Comparability.

Selection (maximum 5)

1) Representativeness of the exposed cohort
   a) truly representative of the average in the target population (e.g. random sample or whole population) *
   b) somewhat representative of the average in the target population (e.g. purposive sampling of representative schools or evidence that the sample is representative of the source population) *
   c) selected group, may not be representative of the average in the target population
   d) derivation of the cohort not adequately described

2) Selection of the non-exposed cohort
   a) drawn from the same community as the exposed cohort *
   b) drawn from a different source
   c) no description of the derivation of the non-exposed cohort

3) Ascertainment of exposure (depression)
   a) standardised diagnostic measure or a named measurement instrument *
   b) reported by school, parents or other collateral
   c) no description

4) Adjustment made for baseline or prior educational attainment in multivariable analysis
   a) yes *
   b) no

5) Sample size
   a) Justified and satisfactory *
   b) Not justified

Comparability (maximum 2)

1) Comparability of cohorts on the basis of the design or analysis
   a) study controls for age and gender (either in selection of the cohort, or in adjusted/stratified analyses) *
   b) study controls for any additional factor *
   c) study does not control for any factors

Outcome (maximum 4)

1) Assessment of outcome (educational attainment)
   a) school or administrative records *
   b) self-report
   c) reported by school staff, parents or other collateral
   d) no description

For peer review only

2) Was follow-up long enough for outcomes to occur
   a) yes (≥1 year) *
   b) no

3) Adequacy of follow up of cohorts
   a) complete follow up - all subjects accounted for *
   b) subjects lost to follow up unlikely to introduce bias - small number lost (<20%) or attrition described and accounted for in analysis *
   c) follow up rate inadequate (>20%) and no description of those lost or accounting for those lost in analysis
   d) no statement

4) Statistical test
   a) The statistical test(s) used to analyse the data is clearly described, appropriate and complete, including a named effect estimate, a p value, and a measure of precision if appropriate (i.e. standard errors for beta coefficients, confidence intervals for odds ratios) *
   b) The statistical test is not appropriate, not described or incomplete

For peer review only

BMJ Open

The association between depression and later educational attainment in children and adolescents: A systematic review protocol

Journal:	*BMJ Open*
Manuscript ID	bmjopen-2019-031595.R1
Article Type:	Protocol
Date Submitted by the Author:	03-Sep-2019
Complete List of Authors:	Wickersham, Alice; King's College London, Institute of Psychiatry, Psychology and Neuroscience;<br>Epstein, Sophie; South London and Maudsley NHS Foundation Trust, NIHR Maudsley Biomedical Research Centre<br>Sugg, Holly; University of Exeter, Medical School<br>Stewart, Robert; King's College London, Institute of Psychiatry<br>Ford, Tamsin; University of Exeter<br>Downs, Johnny; Kings College London, Institute of Psychiatry, Psychology and Neuroscience
**Primary Subject Heading**:	Mental health
Secondary Subject Heading:	Epidemiology
Keywords:	Child & adolescent psychiatry < PSYCHIATRY, Depression & mood disorders < PSYCHIATRY, EPIDEMIOLOGY, EDUCATION & TRAINING (see Medical Education & Training)

SCHOLARONE™
Manuscripts

The association between depression and later educational attainment in children and adolescents: A systematic review protocol

Alice Wickersham[1,2], Sophie Epstein[1,2], Holly VR Sugg[3], Robert Stewart[1,2], Tamsin Ford[3] & Johnny Downs[1,2]

[1] Institute of Psychiatry, Psychology & Neuroscience, King's College London, London, UK

[2] NIHR Maudsley Biomedical Research Centre, South London and Maudsley NHS Foundation Trust, London, UK

[3] University of Exeter Medical School, Exeter, UK

Correspondence to:

Alice Wickersham

ORCID ID: 0000-0002-7402-7690

Institute of Psychiatry, Psychology and Neuroscience, King's College London, 16 De Crespigny Park, London, SE5 8AF

alice.wickersham@kcl.ac.uk

020 7848 0002

Word count: 2952 (excluding title page, abstract, references, figures, tables and acknowledgements)

For peer review only

For peer review only - http://bmjopen.bmj.com/site/about/guidelines.xhtml

Abstract

Introduction: Depression represents a major public health concern for children and adolescents, and is thought to negatively impact subsequent educational attainment. However, the extent to which depression and educational attainment are directly associated, and whether other factors play a role, is uncertain. Therefore we aim to systematically review the literature to provide an up-to-date estimate on the strength of this association, and to summarise potential mediators and moderators on the pathway between the two.

Methods and analysis: To identify relevant studies we will systematically search Embase, PsycINFO, PubMed, Education Resources Information Centre and British Education Index, manually search reference lists and contact experts in the field. Studies will be included if they investigate and report on the association between major depression diagnosis or depressive symptoms in children and adolescents aged 4 to 18 years (exposure) and later educational attainment (outcome). Two independent reviewers will screen titles, abstracts and full texts according to eligibility criteria, perform data extraction, and assess study quality according to a modified version of the Newcastle-Ottawa Scale. If sufficiently homogenous studies are identified, summary effect estimates will be pooled in meta-analysis, with further tests for study heterogeneity, publication bias, and the effects of moderators using meta-regression.

Ethics and dissemination: Because this review will make use of already published data, ethical approval will not be sought. The review will be submitted for publication in a peer-reviewed journal, presented at practitioner-facing conferences, and a lay summary will be written for non-scientific audiences such as parents, young people and teachers. The work will inform upcoming investigations on the association between child and adolescent mental health and educational attainment.

PROSPERO registration number: CRD42019123068

Key words: Child & adolescent psychiatry; Depression & mood disorders; Epidemiology; Education & training

ARTICLE SUMMARY

Strengths and limitations of this study

- This review will provide a timely update on the association between depression and later educational attainment, and will be the first to summarise evidence on both moderators and mediators of the association.
- A comprehensive search strategy is planned, including searches of both health and education electronic databases, forward and backward citation searching, and contacting authors and experts in the field.
- The exclusion of grey literature and studies not published in the English language may cause some relevant studies to be missed.
- However, limiting the review to studies which make use of standardised depression measures and academic records will ensure that included studies are of a reasonable quality, and reduce heterogeneity for meta-analysis.
- Including prospective longitudinal studies may also aid inference of a causal direction between depression and later educational attainment.

For peer review only - http://bmjopen.bmj.com/site/about/guidelines.xhtml

INTRODUCTION

Depression is a mental health disorder which, in children and young people, is particularly characterised by symptoms such as low mood, irritability, negative self-perceptions, reduced energy, sleep disturbances and cognitive problems. [1 2] It is a major public health concern for this age group, and appears to be on the rise, with a recent study estimating that 2.1% of children and adolescents in England meet criteria for a depressive disorder. [3-5] Depression is thought to predict a range of negative psychosocial outcomes, including poorer school outcomes such as educational attainment. A meta-analysis of longitudinal studies by Riglin et al. suggests that depression, and to some extent anxiety, are associated with attainment outcomes including the failure to complete compulsory education and low school grades. [6] This may in turn have long-term and far reaching negative consequences, with poor school performance predicting unemployment, homelessness, poor health, and suicide attempt. [7-10]

In spite of this, educational systems have historically shown reluctance to divert resources from traditional teaching towards mental health provision. [11 12] But school-based mental health provision is growing, and the need for a thorough and robust understanding of how depression can impact educational attainment is of critical importance to guide this growing area. [13 14] In particular, a range of candidate factors are thought to affect school performance and may mediate or moderate the impact of depression on educational attainment. Knowledge of these could be used to tailor mental health provision and highlight priority groups for intervention. Candidate factors might include executive function, sleep, classroom environment and engagement. [15-17] Parents and families are also likely to play a critical role in the association, with socio-economic status, the family environment, parent involvement, parental education and parental mental health all thought to be associated with school outcomes. [18-21]

At the time of Riglin et al.'s review, [6] comparatively few studies had been carried out outside of North America. Additionally, while the presence of depressive symptoms remains an exposure of interest, Riglin et al. were unable to draw comparisons of studies to examine the effect of clinical depression on grades, due to an insufficient number of such studies being available. These gaps in the literature may have since been addressed. Therefore, the first aim of our systematic review is to revisit this association and provide an up-to-date estimate on the strength of the relationship between child and adolescent depression and later educational attainment.

Beyond an examination of the moderating effects of age, gender, and length of study follow-up period, Riglin et al. considered evidence on the pathways between depression and educational attainment to be beyond the scope of their systematic review. Therefore, the second aim of our systematic review is to investigate mediators and moderators in the relationship between child and adolescent depression and later educational attainment, with the intention of proposing a pathway model between the two.

METHODS AND ANALYSIS

This protocol follows the PRISMA reporting guidelines for systematic review and meta-analysis protocols (supplementary file 1). [22] The final review will also follow PRISMA reporting guidelines, and will include a PRISMA checklist and flow diagram. [23] Details of the protocol for this systematic review are registered on PROSPERO and can be accessed at www.crd.york.ac.uk/PROSPERO/display_record.asp?ID=CRD42019123068. Any amendments to the protocol will be registered on PROSPERO.

For peer review only - http://bmjopen.bmj.com/site/about/guidelines.xhtml

Eligibility criteria

Table 1: PECOS criteria for inclusion and exclusion of studies

Parameter	Inclusion criteria	Exclusion criteria
**Population**	• Participants aged 4-18 years (inclusive) at the time of exposure measurement<br>• Countries with compulsory education policies	• Participants recruited from post-secondary education settings
**Exposure**	• Depression diagnosis or depressive symptoms as measured using a standardised diagnostic measure or a named measurement instrument	• Internalising symptoms or other affective disorders such as bipolar disorder
**Comparison**	No restrictions	No restrictions
**Outcome**	• Educational attainment as measured using academic or administrative records	• School dropout, general intelligence, aptitude, or ability
**Study design**	• Investigate and report results on the relationship between depression diagnosis or depressive symptoms (exposure) and later educational attainment (outcome)<br>• Quantitative longitudinal studies with prospective data collection<br>• Original research published in a peer-reviewed journal<br>• Published in English<br>• Full-text available including data on the association between depression and educational attainment	• Aim to conduct or evaluate an intervention during the observed study period

Population

Inclusion and exclusion criteria are summarised in Table 1. Because of the relationship under study, we are primarily interested in depression during the school years. Therefore participants in included studies will be children and adolescents, all within the 4 to 18 year age range at the time of exposure measurement, encompassing the compulsory school age range in most countries. Studies will make use of data from countries with compulsory educational policies (as determined from the countries' government or public sector websites) – in countries without such policies, school attendance is likely to be poorer for children with mental health difficulties such as depression, and therefore attainment data is less likely to be adequately available for this group. [24] Studies will be excluded if they recruit participants from post-secondary education settings such as universities at baseline, to ensure that results pertain to depression in childhood and adolescence during school. No further restrictions will be placed on study setting. Age at the time of outcome measurement will not be restricted, such that attainment at higher education completed during adulthood will be included.

Exposure and outcome variables

Included studies will investigate and report results on the relationship between child or adolescent depression (exposure) and later educational attainment (outcome). Child or adolescent depression

For peer review only - http://bmjopen.bmj.com/site/about/guidelines.xhtml

will be operationalised as depressive symptoms or depression diagnosis as identified using a standardised diagnostic measure or a named measurement instrument. Studies of internalising symptoms or other depressive disorders, such as bipolar disorder, will not be included. We anticipate that studies will use various measures of educational attainment, including but not limited to highest level of education completed, and standardised academic assessment scores. Studies will not be included if they do not measure educational attainment using academic or administrative records, instead relying on an unclear source or on self-, parent-, or teacher only-reported outcomes. The accuracy of informant-rated academic performance can be mixed compared to results from externally validated assessments. [25] School dropout will not be considered in this review as it represents a distinct construct which may have different mechanisms associated with it; indeed poor educational attainment is thought to be one of the predictors of school dropout. [26] Measures of general intelligence, aptitude, or ability will also not be included.

Study design

Only quantitative, prospective longitudinal studies will be included. This can aid the inference of a causal direction, particularly where included studies adjust for covariates such as prior attainment. [27 28] Reviews, meta-analyses, cross-sectional studies, retrospective studies, case reports, clinical vignettes, Randomised Controlled Trials, and exclusively qualitative studies will therefore be excluded. Studies which aim to conduct or evaluate an intervention during the observed study period will also be excluded to ensure that any observed association is not influenced by the intervention. Studies will be included if they are original research (therefore excluding editorials, opinion pieces, letters to the editor, and commentaries), are published in English, and are published in a peer-reviewed journal (therefore excluding grey literature, books, chapters, theses, dissertations and conference proceedings). The full text must also be available (including data on the association between depression and educational attainment). Corresponding authors will be contacted for full texts that cannot be obtained publicly or via King's College London's institutional access (two email attempts will be made). To maximise the number of relevant studies captured, no date range will be imposed on the search.

Information sources

Studies will be identified by searching the following electronic databases:

- Embase (via Ovid)
- PsycINFO (via Ovid)
- PubMed (via NCBI)
- Education Resources Information Centre (ERIC) (via EBESCO)
- British Education Index (via EBESCO)

We will also search reference lists of included studies and relevant existing reviews (backward citation searching), and papers which have referenced them (forward citation searching). Backward and forward citation searching will be carried out in Web of Science Core Collection. If a citation cannot be identified in Web of Science, Google Scholar will be searched. Finally, experts in the field and corresponding authors of included studies will be contacted with a link to the PROSPERO record detailing eligibility criteria to identify any additional papers (two email attempts will be made).

Search strategy for electronic databases

Electronic database searching will be conducted using a combination of key words (using truncation as needed) and subject headings (exploded to include narrower terms). The exact search terms used

For peer review only - http://bmjopen.bmj.com/site/about/guidelines.xhtml

will therefore be adapted according to database thesauruses, but broadly will be grouped according to three concepts:

1. Age (e.g. child, adolescent, youth)
2. Educational attainment (e.g. academic performance, educational attainment, school failure)
3. Depression (e.g. depression, depressive disorders)

English language limits will be applied. The full search strategy for Embase can be found in Table 2.

Table 2: Full search strategy for Embase

#	Search terms
1	exp adolescent/ OR exp adolescence/ OR exp child/ OR exp childhood/ OR child*.tw OR adolescen*.tw OR teenag*.tw OR youth*.tw OR (young adj (people or person)).tw
2	Limit 1 to english language
3	exp academic achievement/ OR exp outcome of education/ OR ((academic or educational or school or classroom) adj (achievement or performance or attainment or success or failure)).tw
4	Limit 3 to english language
5	exp depression/ OR depressi*.tw
6	Limit 5 to english language
7	#2 AND #4 AND #6

Note: exp [search term]/ denotes exploding a subject heading; .tw denotes searching for a key word in the title, abstract and drug trade name; * denotes truncation

Data management

All identified citations will be downloaded and managed in EndNote, and duplicates will be removed. Article screening and data extraction will be tracked using Microsoft Excel.

Selection process

Following electronic database searching, initial title and abstract screening will be carried out by two independent reviewers. The reviewers will initially screen 10% of the titles and abstracts and agreement will be checked, before proceeding to screen all the remaining titles and abstracts according to the pre-specified eligibility criteria (Table 1). All references will be screened by both reviewers to ascertain the level of agreement. Articles which appear eligible from the abstract, or are of unclear eligibility, will pass to full-text screening. This will also be carried out by two independent reviewers, again following an initial 10% screen to check agreement. The process of independent abstract and full-text screening will be repeated for references identified during backward and forward citation searching following an initial screen carried out by the lead researcher. Any disagreements over article eligibility will be discussed, and a third reviewer will be consulted if a consensus cannot be reached.

Data extraction

Data will be extracted using a data extraction form which will be informed by the full-text screening and will be piloted on the included studies before being finalised. The anticipated data extraction form is in supplementary file 2. Data extraction will be carried out by two reviewers. Any disagreements over data extraction will be discussed, and a third reviewer will be consulted if a consensus cannot be reached. If multiple studies use the same data sources, they will still be recorded separately in data extraction as they may offer insights to different covariates, mediators and moderators.

For peer review only - http://bmjopen.bmj.com/site/about/guidelines.xhtml

Quality assessment

The included studies will be assessed for risk of bias at the study-level using a modified version of the Newcastle-Ottawa Scale (NOS) for cohort studies. [29] The NOS is recommended by the Cochrane Handbook (section 13.5.2.3). [30] Some items will be adapted for relevance to this review (for instance making reference to depression, educational attainment, and schools), and two items on sample size and statistical tests will be added in accordance with a previous systematic review on education and mental health. [31] Risk of bias will be assessed by two independent reviewers. Risk of bias assessment will be conducted on 10% of the included studies and agreement checked before proceeding to the remaining studies. Disagreements will be discussed, and a third reviewer will be consulted if a consensus cannot be reached. Results from the risk of bias assessment will be taken into consideration when interpreting the strength of evidence for the reported associations, and will be considered in meta-analysis (see 'Data Synthesis').

Data synthesis

The characteristics and findings of included studies will be presented in a data extraction table and will be discussed in a narrative synthesis. If multiple studies are identified which investigate similar exposure and outcome variables, a random-effects meta-analysis will be conducted. If multiple articles measure the same association in an identical cohort, and if multiple relevant associations are reported within one article, the mean of these associations will be taken and used in meta-analysis.

Summary estimates for the effect of depression on later educational attainment will be pooled. Estimates from multivariable analyses which control for at least one covariate will be used, unless insufficient studies conduct comparable multivariable analyses, in which case estimates from bivariate analyses will be used instead. A confidence interval and p value will be calculated for the pooled effect estimate. Heterogeneity of effect estimates will be investigated using the I^2 statistic. Publication bias will be assessed using funnel plots and Egger's test for publication bias if at least ten studies are included in the meta-analysis (Cochrane Handbook section 10.4.3.1). [30]

To investigate moderators in the association between depression and educational attainment, subgroup analyses will be carried out using meta-regression. As with Riglin et al., [6] we will examine the moderating effects of age, gender, and follow-up period. In addition, the impact of risk of bias will be investigated. Other investigations of potentially important moderators may be informed *post hoc* by the included studies, but will be identified as such in the final report as recommended in the Cochrane Handbook (section 9.6.5.2). [30] Candidate moderators include ethnicity, socio-economic status, variables relating to the parent or family context, co-morbidities, country, the measurement of clinical diagnosis versus symptomatology at exposure, and whether studies are conducted in clinical versus community settings. As with the main meta-analysis, this will only be possible where similar moderator variables or analyses are available for multiple studies (for instance, if participant characteristics are measured on similar scales and adjusted for at similar timepoints). Finally, if multiple studies investigate similar mediator variables, meta-analytic structural equation modelling will be employed to synthesise their effects.

Study status

Electronic database searching was conducted in November 2018. The review is expected to be completed in December 2019.

For peer review only - http://bmjopen.bmj.com/site/about/guidelines.xhtml

For peer review only

ETHICS AND DISSEMINATION

This review will make use of already published data, therefore ethical approval will not be sought. On completion, the review will be submitted to a peer-reviewed journal in the field of mental health or educational research for publication. Findings will also be presented at practitioner-facing conferences, and a lay summary will be written for non-scientific audiences such as parents, young people and teachers. The findings will inform upcoming work on the association between child and adolescent mental health and educational attainment.

Patient and public involvement

This review has been planned to support ongoing health and education data linkage work which has been carried out in consultation with several patient and caregiver groups at the National Institute for Health Research Biomedical Research Centre, South London and Maudsley NHS Foundation Trust and King's College London. [32 33] The results of the review and other research will be discussed with a young person's mental health advisory group, parents and teachers to guide upcoming work with linked health and education data.

DISCUSSION

While our methodology is informed by Riglin et al., [6] we have made several modifications to this design which we believe will strengthen the review. First, we will broaden our information sources to include Embase and British Education Index. Second, we will examine the 4 to 18 year age range rather than the 8 to 18 year age range, as this captures the compulsory school years in most countries. Third, we will not limit our review to community-based samples. While findings from clinical samples are of limited generalisability, this will maximise the amount of available evidence that is captured on the association, particularly with regard to diagnosed depressive disorders.

In our review we will focus on depression and will not report data on anxiety or other internalising disorders. Riglin et al. noted that the association between anxiety and educational attainment is much less clear, with the possibility that anxiety may sometimes have a positive role. Therefore, the pathways leading to educational attainment outcomes are likely to be different for anxiety and depression, and should be considered separately. Finally, to ensure the objectivity of our outcome measure, we will exclude studies which do not obtain educational attainment from academic or administrative records.

This review inevitably will have some limitations. Our focus is on attainment subsequent to depression, such that research on the more nuanced relationships between depression and education over time are beyond the scope of this study. Our restriction to longitudinal studies may also result in some relevant data being missed (for instance from case-control studies or the control groups of Randomised Controlled Trials), however this is considered an important inclusion criteria as longitudinal designs are well suited for investigating the relationships under study and can aid causal inference. [27] The exclusion of studies not published in the English language may omit some studies of this association, however we lack resources for translation. Additionally, the exclusion of grey literature may also cause relevant findings to be missed.

This systematic review will provide a synthesis of the available evidence on the association between child and adolescent depression and later educational attainment. This work is timely and of great public interest. Recent studies demonstrate the high prevalence of mental health problems in the child and adolescent age group, [3-5] and ongoing plans to improve child mental health provision in

For peer review only - http://bmjopen.bmj.com/site/about/guidelines.xhtml

schools demonstrate the increasing recognition that education and wellbeing are closely linked. [13] Understanding whether and how depression influences later educational attainment is critical to developing effective interventions for affected groups. To our knowledge this will be the first systematic review to provide a focused synthesis on child and adolescent depression and academic records of educational attainment, with the additional aim of investigating mediators and moderators in order to propose a comprehensive pathway model on the association between the two.

Author contributions: AW (guarantor) co-conceived and designed the review, developed the search strategy, and wrote the initial manuscript. SE advised on study design and provided revisions on the manuscript. HVRS provided revisions on the manuscript. RS, TF and JD co-conceived the review, advised on study design, and provided revisions on the manuscript.

Acknowledgements: With thanks to Simon Benham-Clarke and Darren Moore for providing comments on the search strategy, and to Aida Bonham for assisting during the early stages of review design.

Competing Interests: None declared

Funding statement: This paper represents independent research funded by the National Institute for Health Research (NIHR) Biomedical Research Centre at South London and Maudsley NHS Foundation Trust and King's College London. The views expressed are those of the authors and not necessarily those of the NHS, the NIHR or the Department of Health and Social Care. SE has been employed as an NIHR Academic Clinical Fellow and has received salary support from an MQ Data Science Award and from the Psychiatry Research Trust. HVRS is funded by the University of Exeter Medical School. JD is funded by an NIHR Clinician Scientist Award, and received support from a Medical Research Council (MRC) Clinical Research Training Fellowship (MR/L017105/1) and Psychiatry Research Trust Peggy Pollak Research Fellowship in Developmental Psychiatry.

Data Availability Statement: Not applicable

For peer review only

For peer review only - http://bmjopen.bmj.com/site/about/guidelines.xhtml

References

1. Nardi B, Francesconi G, Catena-Dell'osso M, et al. Adolescent depression: clinical features and therapeutic strategies. *Eur Rev Med Pharmacol Sci* 2013;17(11):1546-51.
2. Orchard F, Pass L, Marshall T, et al. Clinical characteristics of adolescents referred for treatment of depressive disorders. *Child and Adolescent Mental Health* 2017;22(2):61-68. doi: 10.1111/camh.12178
3. Lewis SJ, Arseneault L, Caspi A, et al. The epidemiology of trauma and post-traumatic stress disorder in a representative cohort of young people in England and Wales. *The Lancet Psychiatry* 2019;6(3):247-56. doi: 10.1016/S2215-0366(19)30031-8
4. Pitchforth J, Fahy K, Ford T, et al. Mental health and well-being trends among children and young people in the UK, 1995–2014: analysis of repeated cross-sectional national health surveys. *Psychological medicine* 2018:1-11.
5. Sadler K, Vizard T, Ford T, et al. Mental Health of Children and Young People in England, 2017. 2018
6. Riglin L, Petrides K, Frederickson N, et al. The relationship between emotional problems and subsequent school attainment: A meta-analysis. *Journal of adolescence* 2014;37(4):335-46.
7. Almquist YB. School performance as a precursor of adult health: exploring associations to disease-specific hospital care and their possible explanations. *Scandinavian journal of public health* 2013;41(1):81-91.
8. Brakenhoff B, Jang B, Slesnick N, et al. Longitudinal predictors of homelessness: Findings from the National Longitudinal Survey of Youth-97. *Journal of youth studies* 2015;18(8):1015-34.
9. Caspi A, Wright BRE, Moffitt TE, et al. Early failure in the labor market: Childhood and adolescent predictors of unemployment in the transition to adulthood. *American sociological review* 1998:424-51.
10. Sörberg Wallin A, Zeebari Z, Lager A, et al. Suicide attempt predicted by academic performance and childhood IQ: a cohort study of 26 000 children. *Acta psychiatrica scandinavica* 2018;137(4):277-86.
11. Bonell C, Humphrey N, Fletcher A, et al. Why schools should promote students' health and wellbeing. *BMJ : British Medical Journal* 2014;348:g3078. doi: 10.1136/bmj.g3078
12. Humphrey N, Wigelsworth M. Making the case for universal school-based mental health screening. *Emotional and Behavioural Difficulties* 2016;21(1):22-42.
13. Department of Health and Social Care and Department for Education. Transforming children and young people's mental health provision: A green paper: DoH/DfE London, 2017.
14. Werner-Seidler A, Perry Y, Calear AL, et al. School-based depression and anxiety prevention programs for young people: A systematic review and meta-analysis. *Clinical Psychology Review* 2017;51:30-47. doi: https://doi.org/10.1016/j.cpr.2016.10.005
15. Dewald JF, Meijer AM, Oort FJ, et al. The influence of sleep quality, sleep duration and sleepiness on school performance in children and adolescents: A meta-analytic review. *Sleep medicine reviews* 2010;14(3):179-89.
16. Diamantopoulou S, Rydell A-M, Thorell LB, et al. Impact of executive functioning and symptoms of attention deficit hyperactivity disorder on children's peer relations and school performance. *Developmental neuropsychology* 2007;32(1):521-42.
17. Dotterer AM, Lowe K. Classroom context, school engagement, and academic achievement in early adolescence. *Journal of youth and adolescence* 2011;40(12):1649-60.
18. Augustine JM, Crosnoe R. Mothers' depression and educational attainment and their children's academic trajectories. *Journal of health and social behavior* 2010;51(3):274-90. doi: 10.1177/0022146510377757
19. Harwell M, Maeda Y, Bishop K, et al. The Surprisingly Modest Relationship Between SES and Educational Achievement. *The Journal of Experimental Education* 2017;85(2):197-214. doi: 10.1080/00220973.2015.1123668

For peer review only - http://bmjopen.bmj.com/site/about/guidelines.xhtml

20. Marjoribanks K. Family environments and children's outcomes. *Educational Psychology* 2005;25(6):647-57.
21. Wilder S. Effects of parental involvement on academic achievement: A meta-synthesis. *Educational Review* 2014;66(3):377-97.
22. Shamseer L, Moher D, Clarke M, et al. Preferred reporting items for systematic review and meta-analysis protocols (PRISMA-P) 2015: elaboration and explanation. *Bmj* 2015;349:g7647.
23. Moher D, Liberati A, Tetzlaff J, et al. Preferred reporting items for systematic reviews and meta-analyses: the PRISMA statement. *Annals of internal medicine* 2009;151(4):264-69.
24. Quan A. Education for people and planet: Creating sustainable futures for all: Unesco 2016.
25. Kuncel NR, Credé M, Thomas LL. The validity of self-reported grade point averages, class ranks, and test scores: A meta-analysis and review of the literature. *Review of educational research* 2005;75(1):63-82.
26. De Witte K, Cabus S, Thyssen G, et al. A critical review of the literature on school dropout. *Educational Research Review* 2013;10:13-28.
27. Parab S, Bhalerao S. Study designs. *Int J Ayurveda Res* 2010;1(2):128-31. doi: 10.4103/0974-7788.64406
28. VanderWeele TJ, Jackson JW, Li S. Causal inference and longitudinal data: a case study of religion and mental health. *Social Psychiatry and Psychiatric Epidemiology* 2016;51(11):1457-66. doi: 10.1007/s00127-016-1281-9
29. Wells GA, Shea, B., O'Connell, D., Peterson, J., Welch, V., Losos, M., Tugwell, P. The Newcastle-Ottawa Scale (NOS) for assessing the quality of nonrandomised studies in meta-analyses 2008 [Available from: http://www.ohri.ca/programs/clinical_epidemiology/oxford.asp.
30. Higgins J, Green S. Cochrane Handbook for Systematic Reviews of Interventions Version 5.1.0 [updated March 2011]: The Cochrane Collaboration 2011.
31. Epstein S, Roberts E, Sedgwick R, et al. Poor school attendance and exclusion: a systematic review protocol on educational risk factors for self-harm and suicidal behaviours. *BMJ Open* 2018;8(12):e023953. doi: 10.1136/bmjopen-2018-023953
32. Downs J, Gilbert R, Hayes RD, et al. Linking health and education data to plan and evaluate services for children. *Archives of disease in childhood* 2017;102(7):599-602.
33. Downs JM, Ford T, Stewart R, et al. An approach to linking education, social care and electronic health records for children and young people in South London: a linkage study of child and adolescent mental health service data. *BMJ Open* 2019;9(1):e024355. doi: 10.1136/bmjopen-2018-024355

For peer review only

For peer review only - http://bmjopen.bmj.com/site/about/guidelines.xhtml

Supplementary file 1 – PRISMA-P checklist

PRISMA-P (Preferred Reporting Items for Systematic review and Meta-Analysis Protocols) 2015 checklist: recommended items to address in a systematic review protocol*

Section and topic	Item No	Checklist item	Page number
**ADMINISTRATIVE INFORMATION**			
Title:			
Identification	1a	Identify the report as a protocol of a systematic review	1
Update	1b	If the protocol is for an update of a previous systematic review, identify as such	N/A
Registration	2	If registered, provide the name of the registry (such as PROSPERO) and registration number	2, 3
Authors:			
Contact	3a	Provide name, institutional affiliation, e-mail address of all protocol authors; provide physical mailing address of corresponding author	1
Contributions	3b	Describe contributions of protocol authors and identify the guarantor of the review	9
Amendments	4	If the protocol represents an amendment of a previously completed or published protocol, identify as such and list changes; otherwise, state plan for documenting important protocol amendments	N/A
Support:			
Sources	5a	Indicate sources of financial or other support for the review	9
Sponsor	5b	Provide name for the review funder and/or sponsor	9
Role of sponsor or funder	5c	Describe roles of funder(s), sponsor(s), and/or institution(s), if any, in developing the protocol	N/A
**INTRODUCTION**			
Rationale	6	Describe the rationale for the review in the context of what is already known	3
Objectives	7	Provide an explicit statement of the question(s) the review will address with reference to participants, interventions, comparators, and outcomes (PICO)	3
**METHODS**			
Eligibility criteria	8	Specify the study characteristics (such as PICO, study design, setting, time frame) and report characteristics (such as years considered, language, publication status) to be used as criteria for eligibility for the review	3, 4, 5

For peer review only

Information sources	9	Describe all intended information sources (such as electronic databases, contact with study authors, trial registers or other grey literature sources) with planned dates of coverage	5
Search strategy	10	Present draft of search strategy to be used for at least one electronic database, including planned limits, such that it could be repeated	5, 6
Study records:			
Data management	11a	Describe the mechanism(s) that will be used to manage records and data throughout the review	6
Selection process	11b	State the process that will be used for selecting studies (such as two independent reviewers) through each phase of the review (that is, screening, eligibility and inclusion in meta-analysis)	6
Data collection process	11c	Describe planned method of extracting data from reports (such as piloting forms, done independently, in duplicate), any processes for obtaining and confirming data from investigators	6
Data items	12	List and define all variables for which data will be sought (such as PICO items, funding sources), any pre-planned data assumptions and simplifications	6, supplementary file 2
Outcomes and prioritization	13	List and define all outcomes for which data will be sought, including prioritization of main and additional outcomes, with rationale	3, 4
Risk of bias in individual studies	14	Describe anticipated methods for assessing risk of bias of individual studies, including whether this will be done at the outcome or study level, or both; state how this information will be used in data synthesis	7
Data synthesis	15a	Describe criteria under which study data will be quantitatively synthesised	7
	15b	If data are appropriate for quantitative synthesis, describe planned summary measures, methods of handling data and methods of combining data from studies, including any planned exploration of consistency (such as I^2, Kendall's τ)	7
	15c	Describe any proposed additional analyses (such as sensitivity or subgroup analyses, meta-regression)	7
	15d	If quantitative synthesis is not appropriate, describe the type of summary planned	7
Meta-bias(es)	16	Specify any planned assessment of meta-bias(es) (such as publication bias across studies, selective reporting within studies)	7
Confidence in cumulative evidence	17	Describe how the strength of the body of evidence will be assessed (such as GRADE)	7

*** It is strongly recommended that this checklist be read in conjunction with the PRISMA-P Explanation and Elaboration (cite when available) for important clarification on the items. Amendments to a review protocol should be tracked and dated. The copyright for PRISMA-P (including checklist) is held by the PRISMA-P Group and is distributed under a Creative Commons Attribution Licence 4.0.**

From: Shamseer L, Moher D, Clarke M, Ghersi D, Liberati A, Petticrew M, Shekelle P, Stewart L, PRISMA-P Group. Preferred reporting items for systematic review and meta-analysis protocols (PRISMA-P) 2015: elaboration and explanation. BMJ. 2015 Jan 2;349(jan02 1):g7647.

For peer review only

Supplementary file 2 – Data extraction form

- Lead author
- Year
- Country
- Participant inclusion/exclusion criteria
- Sample size for reported analysis
- Sample type (clinical/community/school)
- Age at exposure (mean, median and/or range)
- Follow-up period
- Gender balance
- Exposure type (binary/continuous/categorical)
- Exposure method of ascertainment
- Outcome type (binary/continuous/categorical)
- Outcome method of ascertainment
- Multivariable or bivariate effect estimate
- Confidence intervals or standard error
- p value
- Covariates and confounders adjusted for
- Moderators reported on
- Moderator findings
- Mediators reported on
- Mediator findings

For peer review only

BMJ Open

The association between depression and later educational attainment in children and adolescents: A systematic review protocol

Journal:	*BMJ Open*
Manuscript ID	bmjopen-2019-031595.R2
Article Type:	Protocol
Date Submitted by the Author:	11-Oct-2019
Complete List of Authors:	Wickersham, Alice; King's College London, Institute of Psychiatry, Psychology and Neuroscience;<br>Epstein, Sophie; South London and Maudsley NHS Foundation Trust, NIHR Maudsley Biomedical Research Centre<br>Sugg, Holly; University of Exeter, Medical School<br>Stewart, Robert; King's College London, Institute of Psychiatry<br>Ford, Tamsin; University of Exeter<br>Downs, Johnny; Kings College London, Institute of Psychiatry, Psychology and Neuroscience
**Primary Subject Heading**:	Mental health
Secondary Subject Heading:	Epidemiology
Keywords:	Child & adolescent psychiatry < PSYCHIATRY, Depression & mood disorders < PSYCHIATRY, EPIDEMIOLOGY, EDUCATION & TRAINING (see Medical Education & Training)

SCHOLARONE™
Manuscripts

The association between depression and later educational attainment in children and adolescents: A systematic review protocol

Alice Wickersham[1,2], Sophie Epstein[1,2], Holly VR Sugg[3], Robert Stewart[1,2], Tamsin Ford[3] & Johnny Downs[1,2]

[1] Institute of Psychiatry, Psychology & Neuroscience, King's College London, London, UK

[2] NIHR Maudsley Biomedical Research Centre, South London and Maudsley NHS Foundation Trust, London, UK

[3] University of Exeter Medical School, Exeter, UK

Correspondence to:

Alice Wickersham

ORCID ID: 0000-0002-7402-7690

Institute of Psychiatry, Psychology and Neuroscience, King's College London, 16 De Crespigny Park, London, SE5 8AF

alice.wickersham@kcl.ac.uk

020 7848 0002

Word count: 2873 (excluding title page, abstract, references, figures, tables and acknowledgements)

For peer review only

For peer review only - http://bmjopen.bmj.com/site/about/guidelines.xhtml

Abstract

Introduction: Depression represents a major public health concern for children and adolescents, and is thought to negatively impact subsequent educational attainment. However, the extent to which depression and educational attainment are directly associated, and whether other factors play a role, is uncertain. Therefore we aim to systematically review the literature to provide an up-to-date estimate on the strength of this association, and to summarise potential mediators and moderators on the pathway between the two.

Methods and analysis: To identify relevant studies we will systematically search Embase, PsycINFO, PubMed, Education Resources Information Centre and British Education Index, manually search reference lists and contact experts in the field. Studies will be included if they investigate and report on the association between major depression diagnosis or depressive symptoms in children and adolescents aged 4 to 18 years (exposure) and later educational attainment (outcome). Two independent reviewers will screen titles, abstracts and full texts according to eligibility criteria, perform data extraction, and assess study quality according to a modified version of the Newcastle-Ottawa Scale. If sufficiently homogenous studies are identified, summary effect estimates will be pooled in meta-analysis, with further tests for study heterogeneity, publication bias, and the effects of moderators using meta-regression.

Ethics and dissemination: Because this review will make use of already published data, ethical approval will not be sought. The review will be submitted for publication in a peer-reviewed journal, presented at practitioner-facing conferences, and a lay summary will be written for non-scientific audiences such as parents, young people and teachers. The work will inform upcoming investigations on the association between child and adolescent mental health and educational attainment.

PROSPERO registration number: CRD42019123068

Key words: Child & adolescent psychiatry; Depression & mood disorders; Epidemiology; Education & training

ARTICLE SUMMARY

Strengths and limitations of this study

- This review will provide a timely update on the association between depression and later educational attainment, and will be the first to summarise evidence on both moderators and mediators of the association.
- A comprehensive search strategy is planned, including searches of both health and education electronic databases, forward and backward citation searching, and contacting authors and experts in the field.
- The exclusion of grey literature and studies not published in the English language may cause some relevant studies to be missed.
- However, limiting the review to studies which make use of standardised depression measures and academic records will ensure that included studies are of a reasonable quality, and reduce heterogeneity for meta-analysis.
- Including prospective longitudinal studies may also aid inference of a causal direction between depression and later educational attainment.

For peer review only - http://bmjopen.bmj.com/site/about/guidelines.xhtml

INTRODUCTION

Depression is a mental health disorder which, in children and young people, is particularly characterised by symptoms such as low mood, irritability, negative self-perceptions, reduced energy, sleep disturbances and cognitive problems. [1 2] It is a major public health concern for this age group, and appears to be on the rise, with a recent study estimating that 2.1% of children and adolescents in England meet criteria for a depressive disorder. [3-5] Depression is thought to predict a range of negative psychosocial outcomes, including poorer school outcomes such as educational attainment. A meta-analysis of longitudinal studies by Riglin et al. suggests that depression, and to some extent anxiety, are associated with attainment outcomes including the failure to complete compulsory education and low school grades. [6] This may in turn have long-term and far reaching negative consequences, with poor school performance predicting unemployment, homelessness, poor health, and suicide attempt. [7-10]

In spite of this, educational systems have historically shown reluctance to divert resources from traditional teaching towards mental health provision. [11 12] But school-based mental health provision is growing, and the need for a thorough and robust understanding of how depression can impact educational attainment is of critical importance to guide this growing area. [13 14] In particular, a range of candidate factors are thought to affect school performance and may mediate or moderate the impact of depression on educational attainment. Knowledge of these could be used to tailor mental health provision and highlight priority groups for intervention. Candidate factors might include executive function, sleep, classroom environment and engagement. [15-17] Parents and families are also likely to play a critical role in the association, with socio-economic status, the family environment, parent involvement, parental education and parental mental health all thought to be associated with school outcomes. [18-21]

At the time of Riglin et al.'s review, [6] comparatively few studies had been carried out outside of North America. Additionally, while the presence of depressive symptoms remains an exposure of interest, Riglin et al. were unable to draw comparisons of studies to examine the effect of clinical depression on grades, due to an insufficient number of such studies being available. These gaps in the literature may have since been addressed. Therefore, the first aim of our systematic review is to revisit this association and provide an up-to-date estimate on the strength of the relationship between child and adolescent depression and later educational attainment.

Beyond an examination of the moderating effects of age, gender, and length of study follow-up period, Riglin et al. considered evidence on the pathways between depression and educational attainment to be beyond the scope of their systematic review. Therefore, the second aim of our systematic review is to investigate mediators and moderators in the relationship between child and adolescent depression and later educational attainment, with the intention of proposing a pathway model between the two.

METHODS AND ANALYSIS

This protocol follows the PRISMA reporting guidelines for systematic review and meta-analysis protocols (supplementary file 1). [22] The final review will also follow PRISMA reporting guidelines, and will include a PRISMA checklist and flow diagram. [23] Details of the protocol for this systematic review are registered on PROSPERO and can be accessed at www.crd.york.ac.uk/PROSPERO/display_record.asp?ID=CRD42019123068. Any amendments to the protocol will be registered on PROSPERO.

For peer review only - http://bmjopen.bmj.com/site/about/guidelines.xhtml

Eligibility criteria

Table 1: PECOS criteria for inclusion and exclusion of studies

Parameter	Inclusion criteria	Exclusion criteria
**Population**	• Participants aged 4-18 years (inclusive) at the time of exposure measurement<br>• Countries with compulsory education policies	• Participants recruited from post-secondary education settings
**Exposure**	• Depression diagnosis or depressive symptoms as measured using a standardised diagnostic measure or a named measurement instrument	• Internalising symptoms or other affective disorders such as bipolar disorder
**Comparison**	No restrictions	No restrictions
**Outcome**	• Educational attainment as measured using academic or administrative records	• School dropout, general intelligence, aptitude, or ability
**Study design**	• Investigate and report results on the relationship between depression diagnosis or depressive symptoms (exposure) and later educational attainment (outcome)<br>• Quantitative longitudinal studies with prospective data collection<br>• Original research published in a peer-reviewed journal<br>• Published in English<br>• Full-text available including data on the association between depression and educational attainment	• Aim to conduct or evaluate an intervention during the observed study period

Population

Inclusion and exclusion criteria are summarised in Table 1. Because of the relationship under study, we are primarily interested in depression during the school years. Therefore participants in included studies will be children and adolescents, all within the 4 to 18 year age range at the time of exposure measurement, encompassing the compulsory school age range in most countries. Studies will make use of data from countries with compulsory educational policies (as determined from the countries' government or public sector websites) – in countries without such policies, school attendance is likely to be poorer for children with mental health difficulties such as depression, and therefore attainment data is less likely to be adequately available for this group. [24] Studies will be excluded if they recruit participants from post-secondary education settings such as universities at baseline, to ensure that results pertain to depression in childhood and adolescence during school. No further restrictions will be placed on study setting. Age at the time of outcome measurement will not be restricted, such that attainment at higher education completed during adulthood will be included.

Exposure and outcome variables

Included studies will investigate and report results on the relationship between child or adolescent depression (exposure) and later educational attainment (outcome). Child or adolescent depression

For peer review only - http://bmjopen.bmj.com/site/about/guidelines.xhtml

will be operationalised as depressive symptoms or depression diagnosis as identified using a standardised diagnostic measure or a named measurement instrument. Studies of internalising symptoms or other depressive disorders, such as bipolar disorder, will not be included. We anticipate that studies will use various measures of educational attainment, including but not limited to highest level of education completed, and standardised academic assessment scores. Studies will not be included if they do not measure educational attainment using academic or administrative records, instead relying on an unclear source or on self-, parent-, or teacher only-reported outcomes. The accuracy of informant-rated academic performance can be mixed compared to results from externally validated assessments. [25] School dropout will not be considered in this review as it represents a distinct construct which may have different mechanisms associated with it; indeed poor educational attainment is thought to be one of the predictors of school dropout. [26] Measures of general intelligence, aptitude, or ability will also not be included.

Study design

Only quantitative, prospective longitudinal studies will be included. This can aid the inference of a causal direction, particularly where included studies adjust for covariates such as prior attainment. [27] [28] Reviews, meta-analyses, cross-sectional studies, retrospective studies, case reports, clinical vignettes, Randomised Controlled Trials, and exclusively qualitative studies will therefore be excluded. Studies which aim to conduct or evaluate an intervention during the observed study period will also be excluded to ensure that any observed association is not influenced by the intervention. Studies will be included if they are original research (therefore excluding editorials, opinion pieces, letters to the editor, and commentaries), are published in English, and are published in a peer-reviewed journal (therefore excluding grey literature, books, chapters, theses, dissertations and conference proceedings). The full text must also be available (including data on the association between depression and educational attainment). Corresponding authors will be contacted for full texts that cannot be obtained publicly or via King's College London's institutional access (two email attempts will be made). To maximise the number of relevant studies captured, no date range will be imposed on the search.

Information sources

Studies will be identified by searching the following electronic databases:

- Embase (via Ovid)
- PsycINFO (via Ovid)
- PubMed (via NCBI)
- Education Resources Information Centre (ERIC) (via EBESCO)
- British Education Index (via EBESCO)

We will also search reference lists of included studies and relevant existing reviews (backward citation searching), and papers which have referenced them (forward citation searching). Backward and forward citation searching will be carried out in Web of Science Core Collection. If a citation cannot be identified in Web of Science, Google Scholar will be searched. Finally, experts in the field and corresponding authors of included studies will be contacted with a link to the PROSPERO record detailing eligibility criteria to identify any additional papers (two email attempts will be made).

Search strategy for electronic databases

Electronic database searching will be conducted using a combination of key words (using truncation as needed) and subject headings (exploded to include narrower terms). The exact search terms used

For peer review only - http://bmjopen.bmj.com/site/about/guidelines.xhtml

For peer review only

will therefore be adapted according to database thesauruses, but broadly will be grouped according to three concepts:

1. Age (e.g. child, adolescent, youth)
2. Educational attainment (e.g. academic performance, educational attainment, school failure)
3. Depression (e.g. depression, depressive disorders)

English language limits will be applied. The full search strategy for Embase can be found in Table 2.

Table 2: Full search strategy for Embase

#	Search terms
1	exp adolescent/ OR exp adolescence/ OR exp child/ OR exp childhood/ OR child*.tw OR adolescen*.tw OR teenag*.tw OR youth*.tw OR (young adj (people or person)).tw
2	Limit 1 to english language
3	exp academic achievement/ OR exp outcome of education/ OR ((academic or educational or school or classroom) adj (achievement or performance or attainment or success or failure)).tw
4	Limit 3 to english language
5	exp depression/ OR depressi*.tw
6	Limit 5 to english language
7	#2 AND #4 AND #6

Note: exp [search term]/ denotes exploding a subject heading; .tw denotes searching for a key word in the title, abstract and drug trade name; * denotes truncation

Data management

All identified citations will be downloaded and managed in EndNote, and duplicates will be removed. Article screening and data extraction will be tracked using Microsoft Excel.

Selection process

Following electronic database searching, initial title and abstract screening will be carried out by two independent reviewers. The reviewers will initially screen 10% of the titles and abstracts and agreement will be checked, before proceeding to screen all the remaining titles and abstracts according to the pre-specified eligibility criteria (Table 1). All references will be screened by both reviewers to ascertain the level of agreement. Articles which appear eligible from the abstract, or are of unclear eligibility, will pass to full-text screening. This will also be carried out by two independent reviewers, again following an initial 10% screen to check agreement. The process of independent abstract and full-text screening will be repeated for references identified during backward and forward citation searching following an initial screen carried out by the lead researcher. Any disagreements over article eligibility will be discussed, and a third reviewer will be consulted if a consensus cannot be reached.

Data extraction

Data will be extracted using a data extraction form which will be informed by the full-text screening and will be piloted on the included studies before being finalised. The anticipated data extraction form is in supplementary file 2. Data extraction will be carried out by two reviewers. Any disagreements over data extraction will be discussed, and a third reviewer will be consulted if a consensus cannot be reached. If multiple studies use the same data sources, they will still be recorded separately in data extraction as they may offer insights to different covariates, mediators and moderators.

For peer review only - http://bmjopen.bmj.com/site/about/guidelines.xhtml

Quality assessment

The included studies will be assessed for risk of bias at the study-level using a modified version of the Newcastle-Ottawa Scale (NOS) for cohort studies. [29] The NOS is recommended by the Cochrane Handbook (section 13.5.2.3). [30] Some items will be adapted for relevance to this review (for instance making reference to depression, educational attainment, and schools), and two items on sample size and statistical tests will be added in accordance with a previous systematic review on education and mental health. [31] The comparability of groups with and without depression will primarily be assessed based on whether the study controls for both age and gender, either in the selection of the cohort, or in adjusted or stratified analyses. An additional point will be given to studies which control for any other factor.

Risk of bias will be assessed by two independent reviewers. Risk of bias assessment will be conducted on 10% of the included studies and agreement checked before proceeding to the remaining studies. Disagreements will be discussed, and a third reviewer will be consulted if a consensus cannot be reached. Results from the risk of bias assessment will be taken into consideration when interpreting the strength of evidence for the reported associations, and will be considered in meta-analysis (see 'Data Synthesis').

Data synthesis

The characteristics and findings of included studies will be presented in a data extraction table and will be discussed in a narrative synthesis. If multiple studies are identified which investigate similar exposure and outcome variables, a random-effects meta-analysis will be conducted. If multiple articles measure the same association in an identical cohort, and if multiple relevant associations are reported within one article, the mean of these associations will be taken and used in meta-analysis.

Summary estimates for the effect of depression on later educational attainment will be pooled. Estimates from multivariable analyses which control for at least one covariate will be used, unless insufficient studies conduct comparable multivariable analyses, in which case estimates from bivariate analyses will be used instead. A confidence interval and *p* value will be calculated for the pooled effect estimate. Heterogeneity of effect estimates will be investigated using the I^2 statistic. Publication bias will be assessed using funnel plots and Egger's test for publication bias if at least ten studies are included in the meta-analysis (Cochrane Handbook section 10.4.3.1). [30]

To investigate moderators in the association between depression and educational attainment, subgroup analyses will be carried out using meta-regression. As with Riglin et al., [6] we will examine the moderating effects of age, gender, and follow-up period. In addition, the impact of risk of bias will be investigated. Other investigations of potentially important moderators may be informed *post hoc* by the included studies, but will be identified as such in the final report as recommended in the Cochrane Handbook (section 9.6.5.2). [30] Candidate moderators include ethnicity, socio-economic status, variables relating to the parent or family context, co-morbidities, country, the measurement of clinical diagnosis versus symptomatology at exposure, and whether studies are conducted in clinical versus community settings. As with the main meta-analysis, this will only be possible where similar moderator variables or analyses are available for multiple studies (for instance, if participant characteristics are measured on similar scales and adjusted for at similar timepoints). Finally, if multiple studies investigate similar mediator variables, meta-analytic structural equation modelling will be employed to synthesise their effects.

For peer review only - http://bmjopen.bmj.com/site/about/guidelines.xhtml

Study status

Initial electronic database searching was conducted in November 2018. The search will be updated prior to completion, with the review expected to be completed in December 2019.

ETHICS AND DISSEMINATION

This review will make use of already published data, therefore ethical approval will not be sought. On completion, the review will be submitted to a peer-reviewed journal in the field of mental health or educational research for publication. Findings will also be presented at practitioner-facing conferences, and a lay summary will be written for non-scientific audiences such as parents, young people and teachers. The findings will inform upcoming work on the association between child and adolescent mental health and educational attainment.

Patient and public involvement

This review has been planned to support ongoing health and education data linkage work which has been carried out in consultation with several patient and caregiver groups at the National Institute for Health Research Biomedical Research Centre, South London and Maudsley NHS Foundation Trust and King's College London. [32 33] The results of the review and other research will be discussed with a young person's mental health advisory group, parents and teachers to guide upcoming work with linked health and education data.

DISCUSSION

While our methodology is informed by Riglin et al., [6] we have made several modifications to this design which we believe will strengthen the review. First, we will broaden our information sources to include Embase and British Education Index. Second, we will examine the 4 to 18 year age range rather than the 8 to 18 year age range, as this captures the compulsory school years in most countries. Third, we will not limit our review to community-based samples. While findings from clinical samples are of limited generalisability, this will maximise the amount of available evidence that is captured on the association, particularly with regard to diagnosed depressive disorders.

In our review we will focus on depression and will not report data on anxiety or other internalising disorders. Riglin et al. noted that the association between anxiety and educational attainment is much less clear, with the possibility that anxiety may sometimes have a positive role. Therefore, the pathways leading to educational attainment outcomes are likely to be different for anxiety and depression, and should be considered separately. Finally, to ensure the objectivity of our outcome measure, we will exclude studies which do not obtain educational attainment from academic or administrative records.

This review inevitably will have some limitations. Our focus is on attainment subsequent to depression, such that research on the more nuanced relationships between depression and education over time are beyond the scope of this study. Our restriction to longitudinal studies may also result in some relevant data being missed (for instance from case-control studies or the control groups of Randomised Controlled Trials), however this is considered an important inclusion criteria as longitudinal designs are well suited for investigating the relationships under study and can aid causal inference. [27] The exclusion of studies not published in the English language may omit some

For peer review only - http://bmjopen.bmj.com/site/about/guidelines.xhtml

studies of this association, however we lack resources for translation. Additionally, the exclusion of grey literature may also cause relevant findings to be missed.

This systematic review will provide a synthesis of the available evidence on the association between child and adolescent depression and later educational attainment. This work is timely and of great public interest. Recent studies demonstrate the high prevalence of mental health problems in the child and adolescent age group, [3-5] and ongoing plans to improve child mental health provision in schools demonstrate the increasing recognition that education and wellbeing are closely linked. [13] Understanding whether and how depression influences later educational attainment is critical to developing effective interventions for affected groups. To our knowledge this will be the first systematic review to provide a focused synthesis on child and adolescent depression and academic records of educational attainment, with the additional aim of investigating mediators and moderators in order to propose a comprehensive pathway model on the association between the two.

Author contributions: AW (guarantor) co-conceived and designed the review, developed the search strategy, and wrote the initial manuscript. SE advised on study design and provided revisions on the manuscript. HVRS provided revisions on the manuscript. RS, TF and JD co-conceived the review, advised on study design, and provided revisions on the manuscript.

Acknowledgements: With thanks to Simon Benham-Clarke and Darren Moore for providing comments on the search strategy, and to Aida Bonham for assisting during the early stages of review design.

Competing Interests: None declared

Funding statement: This paper represents independent research funded by the National Institute for Health Research (NIHR) Biomedical Research Centre at South London and Maudsley NHS Foundation Trust and King's College London. The views expressed are those of the authors and not necessarily those of the NHS, the NIHR or the Department of Health and Social Care. SE has been employed as an NIHR Academic Clinical Fellow and has received salary support from an MQ Data Science Award and from the Psychiatry Research Trust. HVRS is funded by the University of Exeter Medical School. J.D. is supported by NIHR Clinician Science Fellowship award (CS-2018-18-ST2-014) and has received support from a Medical Research Council (MRC) Clinical Research Training Fellowship (MR/L017105/1) and Psychiatry Research Trust Peggy Pollak Research Fellowship in Developmental Psychiatry.

Data Availability Statement: Not applicable

For peer review only

References

1. Nardi B, Francesconi G, Catena-Dell'osso M, et al. Adolescent depression: clinical features and therapeutic strategies. *Eur Rev Med Pharmacol Sci* 2013;17(11):1546-51.
2. Orchard F, Pass L, Marshall T, et al. Clinical characteristics of adolescents referred for treatment of depressive disorders. *Child and Adolescent Mental Health* 2017;22(2):61-68. doi: 10.1111/camh.12178
3. Lewis SJ, Arseneault L, Caspi A, et al. The epidemiology of trauma and post-traumatic stress disorder in a representative cohort of young people in England and Wales. *The Lancet Psychiatry* 2019;6(3):247-56. doi: 10.1016/S2215-0366(19)30031-8
4. Pitchforth J, Fahy K, Ford T, et al. Mental health and well-being trends among children and young people in the UK, 1995–2014: analysis of repeated cross-sectional national health surveys. *Psychological medicine* 2018:1-11.
5. Sadler K, Vizard T, Ford T, et al. Mental Health of Children and Young People in England, 2017. 2018
6. Riglin L, Petrides K, Frederickson N, et al. The relationship between emotional problems and subsequent school attainment: A meta-analysis. *Journal of adolescence* 2014;37(4):335-46.
7. Almquist YB. School performance as a precursor of adult health: exploring associations to disease-specific hospital care and their possible explanations. *Scandinavian journal of public health* 2013;41(1):81-91.
8. Brakenhoff B, Jang B, Slesnick N, et al. Longitudinal predictors of homelessness: Findings from the National Longitudinal Survey of Youth-97. *Journal of youth studies* 2015;18(8):1015-34.
9. Caspi A, Wright BRE, Moffitt TE, et al. Early failure in the labor market: Childhood and adolescent predictors of unemployment in the transition to adulthood. *American sociological review* 1998:424-51.
10. Sörberg Wallin A, Zeebari Z, Lager A, et al. Suicide attempt predicted by academic performance and childhood IQ: a cohort study of 26 000 children. *Acta psychiatrica scandinavica* 2018;137(4):277-86.
11. Bonell C, Humphrey N, Fletcher A, et al. Why schools should promote students' health and wellbeing. *BMJ : British Medical Journal* 2014;348:g3078. doi: 10.1136/bmj.g3078
12. Humphrey N, Wigelsworth M. Making the case for universal school-based mental health screening. *Emotional and Behavioural Difficulties* 2016;21(1):22-42.
13. Department of Health and Social Care and Department for Education. Transforming children and young people's mental health provision: A green paper: DoH/DfE London, 2017.
14. Werner-Seidler A, Perry Y, Calear AL, et al. School-based depression and anxiety prevention programs for young people: A systematic review and meta-analysis. *Clinical Psychology Review* 2017;51:30-47. doi: https://doi.org/10.1016/j.cpr.2016.10.005
15. Dewald JF, Meijer AM, Oort FJ, et al. The influence of sleep quality, sleep duration and sleepiness on school performance in children and adolescents: A meta-analytic review. *Sleep medicine reviews* 2010;14(3):179-89.
16. Diamantopoulou S, Rydell A-M, Thorell LB, et al. Impact of executive functioning and symptoms of attention deficit hyperactivity disorder on children's peer relations and school performance. *Developmental neuropsychology* 2007;32(1):521-42.
17. Dotterer AM, Lowe K. Classroom context, school engagement, and academic achievement in early adolescence. *Journal of youth and adolescence* 2011;40(12):1649-60.
18. Augustine JM, Crosnoe R. Mothers' depression and educational attainment and their children's academic trajectories. *Journal of health and social behavior* 2010;51(3):274-90. doi: 10.1177/0022146510377757
19. Harwell M, Maeda Y, Bishop K, et al. The Surprisingly Modest Relationship Between SES and Educational Achievement. *The Journal of Experimental Education* 2017;85(2):197-214. doi: 10.1080/00220973.2015.1123668

For peer review only - http://bmjopen.bmj.com/site/about/guidelines.xhtml

20. Marjoribanks K. Family environments and children's outcomes. *Educational Psychology* 2005;25(6):647-57.
21. Wilder S. Effects of parental involvement on academic achievement: A meta-synthesis. *Educational Review* 2014;66(3):377-97.
22. Shamseer L, Moher D, Clarke M, et al. Preferred reporting items for systematic review and meta-analysis protocols (PRISMA-P) 2015: elaboration and explanation. *Bmj* 2015;349:g7647.
23. Moher D, Liberati A, Tetzlaff J, et al. Preferred reporting items for systematic reviews and meta-analyses: the PRISMA statement. *Annals of internal medicine* 2009;151(4):264-69.
24. Quan A. Education for people and planet: Creating sustainable futures for all: Unesco 2016.
25. Kuncel NR, Credé M, Thomas LL. The validity of self-reported grade point averages, class ranks, and test scores: A meta-analysis and review of the literature. *Review of educational research* 2005;75(1):63-82.
26. De Witte K, Cabus S, Thyssen G, et al. A critical review of the literature on school dropout. *Educational Research Review* 2013;10:13-28.
27. Parab S, Bhalerao S. Study designs. *Int J Ayurveda Res* 2010;1(2):128-31. doi: 10.4103/0974-7788.64406
28. VanderWeele TJ, Jackson JW, Li S. Causal inference and longitudinal data: a case study of religion and mental health. *Social Psychiatry and Psychiatric Epidemiology* 2016;51(11):1457-66. doi: 10.1007/s00127-016-1281-9
29. Wells GA, Shea, B., O'Connell, D., Peterson, J., Welch, V., Losos, M., Tugwell, P. The Newcastle-Ottawa Scale (NOS) for assessing the quality of nonrandomised studies in meta-analyses 2008 [Available from: http://www.ohri.ca/programs/clinical_epidemiology/oxford.asp.
30. Higgins J, Green S. Cochrane Handbook for Systematic Reviews of Interventions Version 5.1.0 [updated March 2011]: The Cochrane Collaboration 2011.
31. Epstein S, Roberts E, Sedgwick R, et al. Poor school attendance and exclusion: a systematic review protocol on educational risk factors for self-harm and suicidal behaviours. *BMJ Open* 2018;8(12):e023953. doi: 10.1136/bmjopen-2018-023953
32. Downs J, Gilbert R, Hayes RD, et al. Linking health and education data to plan and evaluate services for children. *Archives of disease in childhood* 2017;102(7):599-602.
33. Downs JM, Ford T, Stewart R, et al. An approach to linking education, social care and electronic health records for children and young people in South London: a linkage study of child and adolescent mental health service data. *BMJ Open* 2019;9(1):e024355. doi: 10.1136/bmjopen-2018-024355

For peer review only

For peer review only - http://bmjopen.bmj.com/site/about/guidelines.xhtml

Supplementary file 1 – PRISMA-P checklist

PRISMA-P (Preferred Reporting Items for Systematic review and Meta-Analysis Protocols) 2015 checklist: recommended items to address in a systematic review protocol*

Section and topic	Item No	Checklist item	Page number
**ADMINISTRATIVE INFORMATION**			
Title:			
Identification	1a	Identify the report as a protocol of a systematic review	1
Update	1b	If the protocol is for an update of a previous systematic review, identify as such	N/A
Registration	2	If registered, provide the name of the registry (such as PROSPERO) and registration number	2, 3
Authors:			
Contact	3a	Provide name, institutional affiliation, e-mail address of all protocol authors; provide physical mailing address of corresponding author	1
Contributions	3b	Describe contributions of protocol authors and identify the guarantor of the review	9
Amendments	4	If the protocol represents an amendment of a previously completed or published protocol, identify as such and list changes; otherwise, state plan for documenting important protocol amendments	N/A
Support:			
Sources	5a	Indicate sources of financial or other support for the review	9
Sponsor	5b	Provide name for the review funder and/or sponsor	9
Role of sponsor or funder	5c	Describe roles of funder(s), sponsor(s), and/or institution(s), if any, in developing the protocol	N/A
**INTRODUCTION**			
Rationale	6	Describe the rationale for the review in the context of what is already known	3
Objectives	7	Provide an explicit statement of the question(s) the review will address with reference to participants, interventions, comparators, and outcomes (PICO)	3
**METHODS**			
Eligibility criteria	8	Specify the study characteristics (such as PICO, study design, setting, time frame) and report characteristics (such as years considered, language, publication status) to be used as criteria for eligibility for the review	3, 4, 5

For peer review only

Information sources	9	Describe all intended information sources (such as electronic databases, contact with study authors, trial registers or other grey literature sources) with planned dates of coverage	5
Search strategy	10	Present draft of search strategy to be used for at least one electronic database, including planned limits, such that it could be repeated	5, 6
Study records:			
Data management	11a	Describe the mechanism(s) that will be used to manage records and data throughout the review	6
Selection process	11b	State the process that will be used for selecting studies (such as two independent reviewers) through each phase of the review (that is, screening, eligibility and inclusion in meta-analysis)	6
Data collection process	11c	Describe planned method of extracting data from reports (such as piloting forms, done independently, in duplicate), any processes for obtaining and confirming data from investigators	6
Data items	12	List and define all variables for which data will be sought (such as PICO items, funding sources), any pre-planned data assumptions and simplifications	6, supplementary file 2
Outcomes and prioritization	13	List and define all outcomes for which data will be sought, including prioritization of main and additional outcomes, with rationale	3, 4
Risk of bias in individual studies	14	Describe anticipated methods for assessing risk of bias of individual studies, including whether this will be done at the outcome or study level, or both; state how this information will be used in data synthesis	7
Data synthesis	15a	Describe criteria under which study data will be quantitatively synthesised	7
	15b	If data are appropriate for quantitative synthesis, describe planned summary measures, methods of handling data and methods of combining data from studies, including any planned exploration of consistency (such as I^2, Kendall's τ)	7
	15c	Describe any proposed additional analyses (such as sensitivity or subgroup analyses, meta-regression)	7
	15d	If quantitative synthesis is not appropriate, describe the type of summary planned	7
Meta-bias(es)	16	Specify any planned assessment of meta-bias(es) (such as publication bias across studies, selective reporting within studies)	7
Confidence in cumulative evidence	17	Describe how the strength of the body of evidence will be assessed (such as GRADE)	7

*** It is strongly recommended that this checklist be read in conjunction with the PRISMA-P Explanation and Elaboration (cite when available) for important clarification on the items. Amendments to a review protocol should be tracked and dated. The copyright for PRISMA-P (including checklist) is held by the PRISMA-P Group and is distributed under a Creative Commons Attribution Licence 4.0.**

From: Shamseer L, Moher D, Clarke M, Ghersi D, Liberati A, Petticrew M, Shekelle P, Stewart L, PRISMA-P Group. Preferred reporting items for systematic review and meta-analysis protocols (PRISMA-P) 2015: elaboration and explanation. BMJ. 2015 Jan 2;349(jan02 1):g7647.

For peer review only

Supplementary file 2 – Data extraction form

- Lead author
- Year
- Country
- Participant inclusion/exclusion criteria
- Sample size for reported analysis
- Sample type (clinical/community/school)
- Age at exposure (mean, median and/or range)
- Follow-up period
- Gender balance
- Exposure type (binary/continuous/categorical)
- Exposure method of ascertainment
- Outcome type (binary/continuous/categorical)
- Outcome method of ascertainment
- Multivariable or bivariate effect estimate
- Confidence intervals or standard error
- p value
- Covariates and confounders adjusted for
- Moderators reported on
- Moderator findings
- Mediators reported on
- Mediator findings

For peer review only
